# Severe astigmatism secondary to large intraocular lens pantoscopic tilt because of a malpositioned haptic following cataract surgery

**DOI:** 10.1093/jscr/rjae134

**Published:** 2024-03-11

**Authors:** Jonathan T W Au Eong, Jarryl H J Tsai, Kah-Guan Au Eong

**Affiliations:** Lee Kong Chian School of Medicine, Nanyang Technological University, 11 Mandalay Road, 308232, Singapore; Lee Kong Chian School of Medicine, Nanyang Technological University, 11 Mandalay Road, 308232, Singapore; Lee Kong Chian School of Medicine, Nanyang Technological University, 11 Mandalay Road, 308232, Singapore; International Eye Cataract Retina Centre, Mount Elizabeth Medical Centre and Farrer Park Medical Centre, 1 Farrer Park Station Road #14-07/08, Connexion, 217562, Singapore; Department of Ophthalmology and Visual Sciences, Khoo Teck Puat Hospital, 90 Yishun Central, 768828, Singapore

**Keywords:** astigmatism, cataract surgery, intraocular lens, pantoscopic tilt, phacoemulsification

## Abstract

An 80-year-old woman presented with painless blurring of vision and monocular diplopia in her left eye following routine phacoemulsification and monofocal intraocular lens (IOL) implantation 5 weeks earlier. Her uncorrected visual acuity (VA) was 6/60 correctable with pinhole to 6/21. Her best-corrected VA was 6/15 with a subjective refraction of −0.50DS/−5.25DCx37. Her corneal astigmatism was −1.25DCx74. Ophthalmic examination disclosed a severely tilted single-piece posterior chamber IOL in the capsular bag. The inferior portion of the optic was tilted posteriorly because of a twisted and malpositioned haptic. The patient underwent remedial surgery to untwist and reposition the IOL haptic which led to immediate improvement of the IOL position. Her uncorrected VA improved to 6/12^−2^ correctable with pinhole to 12^+1^ with an autorefraction of +0.25DS/−2.00DCx74 on the first postoperative day. One month postoperatively, her best-corrected VA was 6/12 with a refraction of +0.50DS/−2.50DCx82. Her final vision was limited by myopic macular degeneration.

## Introduction

After the initial successful intraocular lens (IOL) implantations by Sir Harold Ridley in the early 1950s [1], IOL implantation has become the gold-standard of visual rehabilitation following cataract removal [2]. Despite progressive advances in surgical technique and IOL design, postoperative complications still exist.

One common complication is the misalignment of IOL, which can occur following complicated or even apparently uneventful cataract surgery [3, 4]. Large amounts of IOL tilt and decentration can have a significant negative effect on visual outcome. In this report, we describe a case of severe IOL pantoscopic tilt causing high astigmatism and impaired vision following routine cataract surgery.

## Case report

An 80-year-old woman presented with painless blurring of vision and monocular diplopia in her left eye following routine phacoemulsification and IOL implantation 5 weeks earlier by a 3rd-year ophthalmology resident in a training hospital. She had uneventful cataract surgery and IOL implantation in her right eye 5 years earlier.

Her uncorrected visual acuity (UCVA) was 6/60 correctable with pinhole to 6/21 in her left eye. Her best-corrected VA (BCVA) was 6/15 with a subjective refraction of −0.50DS/−5.25DCx37. Her corneal astigmatism was −1.25DCx74.

**Figure 1:**
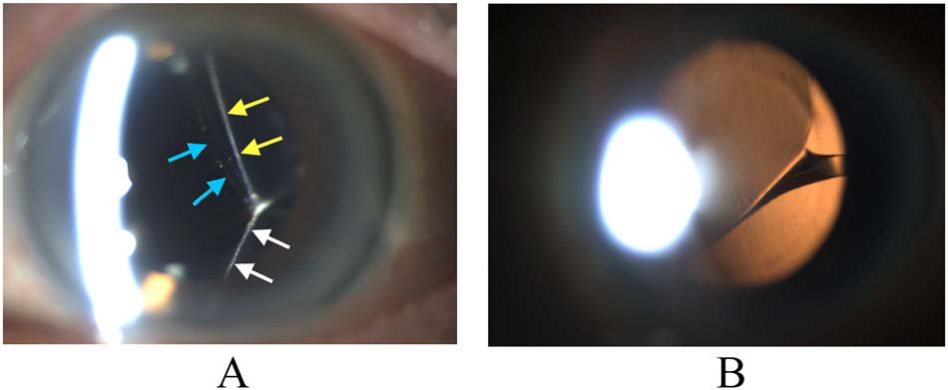
Biomicroscopy of anterior segment on presentation. (A) Illumination using a slit beam coming from the examiner’s left side shows the anterior surface of the IOL (blue arrows) and the posterior capsule superiorly (yellow arrows) and inferiorly (white arrows). The IOL is severely tilted and the posterior capsule is tented backwards by the inferior edge of the IOL. (B) Retroillumination shows IOL malpositioning because of the twisted haptic.

Ophthalmic examination after pupillary dilation revealed a severely tilted Alcon AcrySoft® acrylic foldable single-piece posterior chamber IOL (model SA60AT) in the capsular bag. The anterior capsular rim and posterior capsule were intact. The inferior part of the optic was tilted posteriorly because of a twisted and malpositioned haptic (Fig. 1). The right eye was pseudophakic. The ocular fundus was tessellated and thin on optical coherence tomography because of myopic macular degeneration in both eyes.

The patient underwent remedial surgery to untwist and reposition the haptic, and to re-center the IOL under topical anesthesia. The original temporal clear cornea incision was first reopened using a Sinskey hook. Viscoelastic material (Viscoat®, Alcon) was then injected intracamerally into the anterior chamber. The IOL was then mobilized within the capsular bag using the Sinskey hook. Additional viscoelastic material was then injected posterior to the IOL to separate the IOL from the posterior capsule. Using the Sinskey hook, the optic was manipulated to free the haptic from the equator of the capsule, which allowed the twisted haptic to untwist itself spontaneously. The IOL was then repositioned and centered in the capsular bag. Finally, the viscoelastic material was removed using a Simcoe irrigation/aspiration cannula manually. The corneal wound was self-sealing.

**Figure 2:**
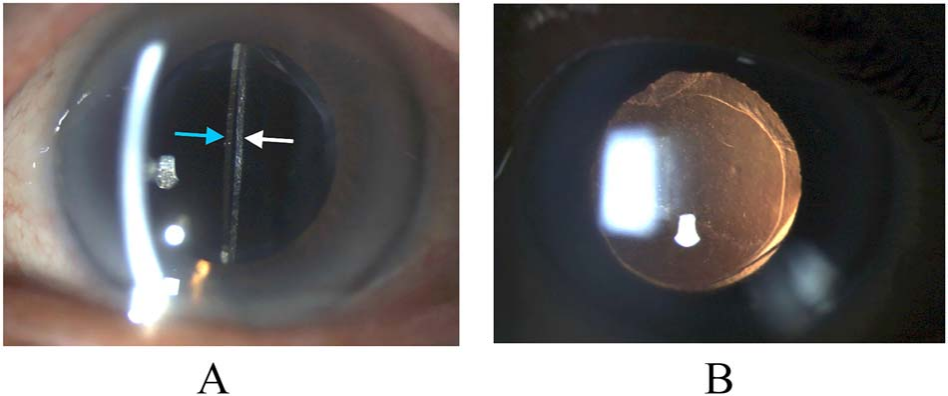
Biomicroscopy of anterior segment at the first postoperative month following remedial surgery. (A) Illumination using a slit beam coming from the examiner’s left side shows the anterior surface of the IOL (blue arrow) and the posterior capsule (white arrow). (B) Retroillumination shows good IOL centration.

Untwisting the haptic and repositioning of the IOL led to immediate improvement of the IOL position (Fig. 2). Her UCVA improved to 6/12^−2^ correctable with pinhole to 6/12^+1^ with an autorefraction of +0.25DS/−2.00DCx74 on the first postoperative day. One month postoperatively, her BCVA was 6/12 with a refraction of +0.50DS/−2.50DCx82. Her final vision was limited by myopic macular degeneration.

## Discussion

IOL misalignment is common, with tilt and decentration greater than 5° and 0.5 mm, respectively, affecting 10% of patients after cataract surgery [5]. It commonly causes visual quality deterioration postoperatively, resulting in glare, astigmatism, visual halo, and/or monocular diplopia [5], some of which were reported by our patient.

IOL tilt and decentration is affected by various factors in the pre-, intra-, and post-operative phases. These include the patient’s preexisting ocular pathology, capsulotomy type, position of the haptics, and IOL design [2, 3, 5]. Small amounts of tilt and decentration often go undetected and are clinically insignificant. Ale *et al*. [2] reported that 2–3° tilt and 0.2–0.3-mm decentration are common and clinically unnoticed for any IOL design. However, tilt of more than 5° or more than 1-mm decentration can cause significant visual loss [5].

Alongside developments in IOL technology and cataract surgery, patient goals have gradually shifted from the simple restoration of sight toward clear vision and reduced visual interference [5]. It is important for surgeons to recognize that IOL tilt and decentration can impact this goal and to try to mitigate this issue, which can be done preoperatively by accurate IOL calculation and selecting appropriate IOL type, and intraoperatively by careful inspection of the IOL centration and haptic positions. Our patient’s severe IOL pantoscopic tilt was not recognized by her surgeon until 30 days later. While postoperative correction of refractive surprise can be achieved through corneal-based surgeries in some cases [6], remedial surgery to untwist and reposition the haptic to achieve better IOL centration appears to be the best approach in our patient.

In summary, refractive surprise after cataract surgery can result from severe IOL pantoscopic tilt from a twisted and malpositioned haptic. While remedial surgery involving haptic and IOL repositioning can reduce the pantoscopic tilt-induced astigmatism, surgeons should carefully check the position of the IOL and its haptics intraoperatively to optimize visual outcomes and avoid additional remedial procedures.
